# Lead exposure is non-linearly associated with subclinical myocardial injury in the general population without cardiovascular disease

**DOI:** 10.3389/fpubh.2022.975413

**Published:** 2022-10-21

**Authors:** Zhenwei Wang, Xu Huang, Jingjie Li, Naifeng Liu, Qin Wei

**Affiliations:** ^1^Department of Cardiology, Zhongda Hospital, School of Medicine, Southeast University, Nanjing, China; ^2^Department of Hematology and Oncology, Affiliated Xuchang People's Hospital of Xinxiang Medical College, Xuchang, China

**Keywords:** environmental pollutants, lead exposure, blood lead, cardiovascular disease, subclinical myocardial injury

## Abstract

**Background and aims:**

Growing studies have focused on the effect of lead exposure on human circulatory system, while the relationship between lead exposure and subclinical myocardial injury (SC-MI) is still poorly known. Therefore, this study was to explore the effect of lead exposure on SC-MI.

**Methods:**

The study included 6,272 individuals aged 40 and older without cardiovascular disease (CVD) from the third National Health and Nutrition Examination Survey. Blood lead was used as an alternative marker of lead exposure. Multivariable logistic regression models, restricted cubic spline and threshold effect analyses were performed to investigate the effect of blood lead on SC-MI.

**Results:**

After adjusting for age, sex, race, diabetes, hypertension, systolic blood pressure, body mass index, waist-to-hip ratio, triglycerides, total cholesterol, creatinine, fasting plasma glucose and hemoglobin Alc, higher blood lead level was independently related to higher risk of SC-MI (OR 1.047, 95% CI [1.018, 1.077]; *P* = 0.003). Restricted cubic spline curve showed that there was a non-linear correlation between blood lead and SC-MI. Threshold effect analysis determined that the inflection point of blood lead was 3.8 ug/dl. When the blood lead level was higher than 3.8 ug/dl, there was an independent positive correlation between blood lead level and the risk of SC-MI (OR 1.031, 95% CI [1.009, 1.053]; *P* < 0.01). And similar associations were also observed among subgroups of male, ≤60 years, >60 years, never smoker, non-Hispanic White, non-Hispanic Black or without hypertension and diabetes.

**Conclusions:**

Blood lead was non-linearly related to SC-MI in population free from CVD.

## Introduction

In the past few decades, cardiovascular disease (CVD) remains one of the leading causes of death in the world, with the total number of cases increasing from 271 million in 1990 to 523 million in 2019, and the number of deaths has increased by nearly 35% during this period, which poses a great health and economic burden (1). Therefore, it is urgent to prevent the occurrence and development of CVD. For all we know, hypertension, dyslipidemia, and diabetes have been perceived as independent risk factors of CVD (1). Nevertheless, with the development of industrialized society, increasing evidence shows that environmental pollutants may also be potential risk factors for CVD (2).

Since the industrial revolution, lead, as the main element involved in environmental pollution, has become a ubiquitous heavy metal in nature. Previous studies have shown that the bioaccumulation of lead in the human body can cause multi-system damage, so lead exposure has become a public health problem of widespread concern (3). Although lead exposure has been well-controlled in the past 20 years with the development of intelligent industry and the improvement of occupational protection (4), several studies have found that long-term low levels of lead exposure can also cause some damage to the health of children and adults (5–7). Lead exposure mainly includes natural exposure (such as contaminated drinking water, food and air, and smoking) and occupational exposure (such as industrial emissions) (8, 9). Lead mainly accumulates in human bone tissue (10), while it is difficult to detect lead in bone, so blood lead is regarded as the most widely used alternative marker of lead exposure (11). As an immunotoxic element, lead can cause many side effects, including hepatotoxicity, nephrotoxicity, endocrine toxicity, immunotoxicity and cardiovascular toxicity, among which the cardiovascular toxicity of lead exposure is the most widely explored (12–16). An increasing number of evidence shows that blood lead is related to circulatory diseases, such as hypertension, peripheral arterial disease, coronary heart disease and stroke (17–20). Additionally, several experimental studies have confirmed the association between blood lead and CVD (21–23). For example, Zeller et al. found that lead could promote arterial intimal hyperplasia and lead to atherosclerosis by endothelial interleukin-8 synthesis mediated by nuclear factor erythroid 2-related factor-2 and subsequent of invasion smooth muscle cells *in vivo* and *vitro* studies (23).

However, we found no any epidemiological studies showing a link between lead exposure and SC-MI. Consequently, this study aimed to evaluate the effect of blood lead on SC-MI in the general population of the United States.

## Materials and methods

### Study population

**Figure 1:**
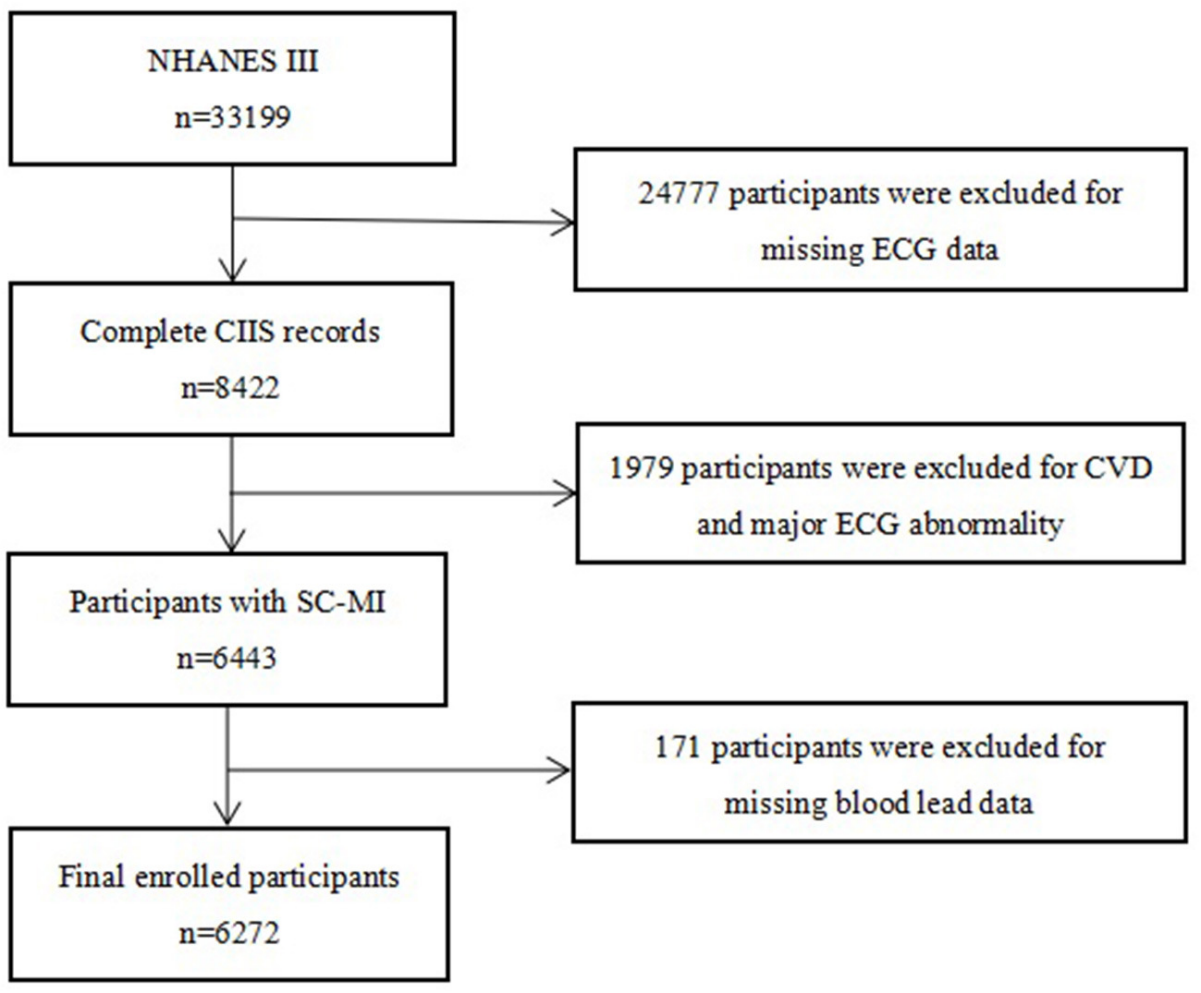
Flow chart of the study population. NHANES, the third National Health and Nutrition Examination Survey; ECG, electrocardiograph; CIIS, cardiac injury/infarction score; CVD, cardiovascular disease; SC-MI, subclinical myocardial injury.

All participants were from third National Health and Nutrition Examination Survey (NHANES III), a nationwide survey involving 33,994 individuals, aimming to assess the nutrition and health status of the general population, the survey design, methods and contents of which were available on NHANES website (https://www.cdc.gov/nchs/nhanes/index.htm). After excluding individuals with CVD and severe abnormal electrocardiograph (ECG), 6,272 participants for whom data were available on blood lead and SC-MI were ultimately enrolled in this study (Figure 1). The protocol of study was approved by the NCHS Ethics Review Board. Written informed consent was provided by all participants, and our study was performed in compliance with the Declaration of Helsinki.

### Analyses of blood lead levels

Blood samples of all participants in this study were collected by professionals when participating in the NHANES III, then stored at −30°C, and finally transported to the NHANES Laboratory of Environmental Health Center of US Centers for Disease Control and Prevention, where they were uniformly measured by professionals. The contaminated and substandard blood samples were removed before determining blood lead. The concentration of lead in blood was determined by graphite furnace atomic absorption spectrophotometry and expressed in ug/dl. Details of the specific determination methods and quality control procedures have been described elsewhere (24).

### Definition of SC-MI

The diagnosis of SC-MI stemmed from a non-invasive, economical and convenient 12-lead ECG-based risk score, that is, cardiac infarction/injury score (CIIS), which was obtained by applying a multivariate decision-theoretic ECG classification scheme and establishing a risk score system reflecting the severity of myocardial injury by experienced people in the light of ECG waveform related to myocardial ischemia, specific details of which were available elsewhere (25). SC-MI was defined as CIIS ≥ 10, without ischemic heart disease and heart failure on the basis of previous study (25, 26).

### Covariates

Demographic information of all participants was obtained by NHANES III investigators through standardized questionnaires, including age, sex, race, diabetes, hypertension, and smoking. In this study, we divided races into non-Hispanic White, non-Hispanic Black, Mexican American, and others. Smoking status was defined as Never, Former or Current (27). Diabetes was defined as having been diagnosed with diabetes by a doctor. Hypertension was defined as having been diagnosed with hypertension by a doctor. Professionals measured blood pressure, body mass index (BMI), and waist-hip ratio (WHR) in all individuals using standard physical examination methods. BMI was defined as weight (kg) divided by the square of height (meter) and expressed in kg/m^2^. WHR was defined as waist circumference (cm) divided by hip circumference (cm). Professional technicians used established experimental procedures to determine the blood parameters of all participants in a standard laboratory, including blood lipids, fasting plasma glucose (FPG), hemoglobin A1c (HbA1c) and creatinine. Details of the specific determination methods and quality control procedures of all covariates were available through NHANES website.

### Statistical analysis

Due to the nature of the multi-stage probability sampling design of NHANES, we adjusted the weights in our analysis to avoid oversampling and reduce the non-response rate, that is, data for continuous and categorical variables were expressed as weighted means (95% CIs) and weighted percentages (95% CIs), respectively. Either a weighted chi-square test (categorical variables) or a weighted linear regression model (continuous variables) were used to calculate differences between groups. And we added the frequency distribution plot on the blood lead stratified by gender (28). Multivariate logistic regression analysis model, restricted cubic spline analysis with 3 knots at 10th, 50th, and 90th percentage and sensitivity analysis were performed to determine the relationship between blood lead and SC-MI. Using R Programming Language (version 3.6.3), SPSS 19.0 (SPSS Inc., Chicago, Illinois, USA) and EmpowerStats (version 2.0) to perform all statistical analyses. A two-tailed *P* < 0.05 was defined as statistically significant.

## Results

### Baseline characteristics of study population

**Figure 2:**
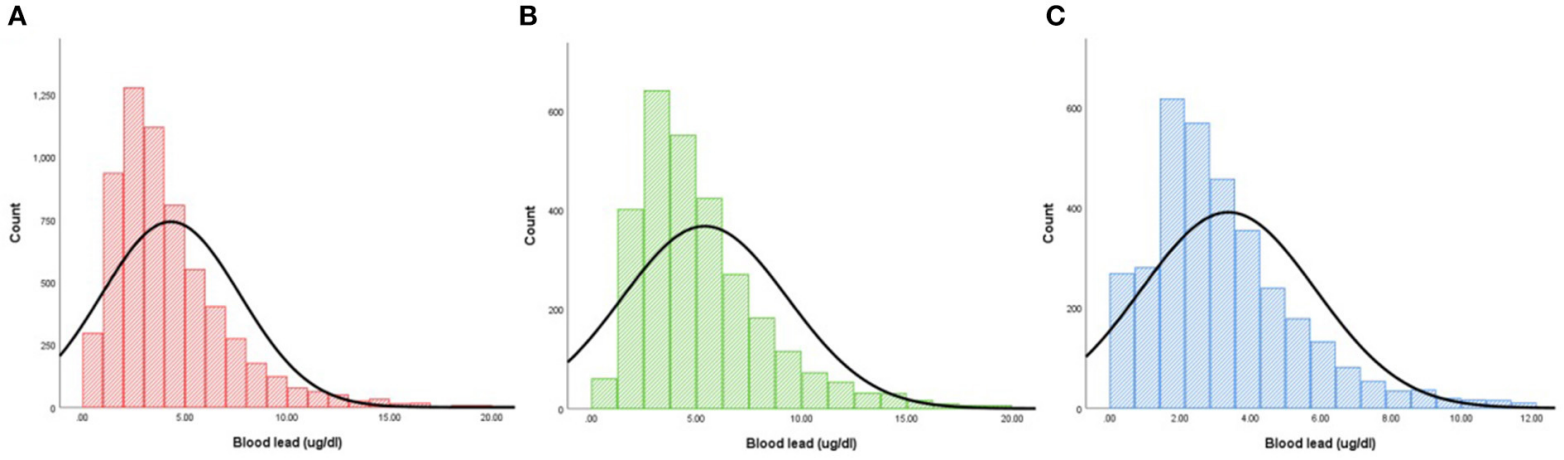
Distribution histogram of blood lead. **(A)** All participants. **(B)** Male. **(C)** Female.

According to CIIS, 6,272 participants (mean age: 58.5 ± 13.1 years old; 46.1% men) were divided into two groups: SC-MI and non-SC-MI group. Individual who suffered from SC-MI tended to be older, smoker and non-Hispanic White, and more likely to develop hypertension and diabetes compared to individual without SC-MI (*P* < 0.001). In terms of traditional risk factors for CVD, participants with SC-MI had higher levels of systolic blood pressure (SBP), WHR, FPG, HbA1c, total cholesterol (TC) and creatinine than those without SC-MI. Importantly, the blood lead levels were also higher in the SC-MI group (Table 1). Figure 2 showed the frequency distribution on the blood lead stratified by gender.

**Table 1:** Baseline characteristics of the participants.

**Variable**	**Overall (*n =* 6,272)**	**SC-MI (*n =* 1,350)**	**Non-SC-MI (*n =* 4,922)**	***P***
Age, years	55.43 (54.69, 56.18)	59.29 (58.00, 60.58)	54.49 (53.79, 55.19)	<0.001
Male, %	44.25 (42.65, 45.86)	44.69 (39.94, 49.54)	44.14 (42.16, 46.14)	0.847
Race/ethnicity, %				<0.001
Non-hispanic white	81.83 (79.32, 84.10)	85.25 (83.24, 87.07)	80.99 (78.18, 83.51)	
Non-hispanic black	7.96 (7.09, 8.94)	8.41 (7.15, 9.85)	7.86 (6.95, 8.87)	
Mexican American	3.52 (3.02, 4.11)	2.96 (2.38, 3.67)	3.66 (3.11, 4.31)	
Others	6.68 (5.04, 8.81)	3.38 (2.35, 4.83)	7.49 (5.62, 9.92)	
Smoking status, %				<0.001
Never	43.37 (41.12, 45.65)	35.26 (31.96, 38.70)	45.37 (42.90, 47.86)	
Former	34.18 (32.44, 35.96)	36.13 (32.96, 39.43)	33.70 (31.72, 35.73)	
Current	22.45 (20.56, 24.45)	35.26 (31.96, 38.70)	20.93 (18.85, 23.18)	
Diabetes, %	6.74 (5.82, 7.80)	10.23 (8.44, 12.34)	5.89 (4.92, 7.02)	<0.001
Hypertension, %	29.63 (27.95, 31.36)	36.03 (32.23, 40.02)	28.05 (26.26, 29.92)	<0.001
Systolic BP, mmHg	127.22 (126.37, 128.07)	130.79 (128.88, 132.71)	126.34 (125.62, 127.05)	<0.001
Diastolic BP, mmHg	76.20 (75.75, 76.66)	75.96 (75.10, 76.83)	76.26 (75.81, 76.71)	0.462
Body mass index, kg/m^2^	27.15 (26.88, 27.41)	27.63 (26.93, 28.34)	27.03 (26.81, 27.25)	0.077
Waist-to-hip ratio	0.93 (0.93, 0.94)	0.94 (0.94, 0.95)	0.93 (0.92, 0.93)	0.002
Triglycerides, mg/dl	157.14 (150.17, 164.10)	165.15 (155.48, 174.82)	155.17 (147.32, 163.02)	0.086
Total cholesterol, mg/dl	217.22 (215.45, 219.00)	221.16 (217.40, 224.92)	216.26 (214.37, 218.14)	0.020
LDL-C, mg/dl	136.80 (134.65, 138.95)	140.48 (136.21, 144.75)	135.91 (133.42, 138.39)	0.077
HDL-C, mg/dl	51.59 (50.70, 52.48)	51.45 (49.97, 52.92)	51.62 (50.70, 52.54)	0.806
Creatinine, mg/dl	1.08 (1.07, 1.09)	1.10 (1.08, 1.12)	1.08 (1.07, 1.08)	0.011
FPG, mg/dl	102.71 (101.38, 104.05)	107.58 (104.30, 110.86)	101.52 (100.06, 102.98)	0.002
Hemoglobin Alc, %	5.53 (5.48, 5.58)	5.72 (5.63, 5.81)	5.48 (5.43, 5.54)	<0.001
Blood lead, ug/dl	3.88 (3.66, 4.10)	4.29 (4.00, 4.57)	3.78 (3.55, 4.01)	<0.001

Data were expressed as weighted mean (95% CI), or weighted percentage (95% CI).

SC-MI, subclinical myocardial injury; BP, blood pressure; LDL-C, low-density lipoprotein cholesterol; HDL-C, high-density lipoprotein cholesterol; FPG, fasting plasma glucose.

### Association between blood lead and SC-MI

The results of multivariate logistic regression analyses for the association between blood lead and SC-MI were showed in Table 2. After adjusting the confounding factors step by step, higher blood lead level was independently related to higher risk of SC-MI in model 3 with adjustment for age, sex, race, smoker, diabetes, hypertension, SBP, BMI, WHR, TG, TC, creatinine, FPG, HbA1c (OR 1.047, 95% CI [1.018, 1.077]; *P* = 0.003).

**Table 2:** Association between blood lead and subclinical myocardial injury.

	**OR**	**95% CI**	***P***
Crude model	1.055	1.028–1.082	<0.001
Model 1	1.041	1.014–1.068	0.004
Model 2	1.022	0.991–1.055	0.161
Model 3	1.047	1.018–1.077	0.003

Crude model: unadjusted. Model 1 was adjusted for age and sex. Model 2 was adjusted for variables included in Model 1 and race, smoking status, diabetes, hypertension. Model 3 was adjusted for variables included in Model 1 and race, diabetes, hypertension, systolic blood pressure, body mass index, waist-to-hip ratio, triglycerides, total cholesterol, creatinine, fasting plasma glucose, hemoglobin Alc. OR, odd ratio; CI, confidence interval.

**Figure 3:**
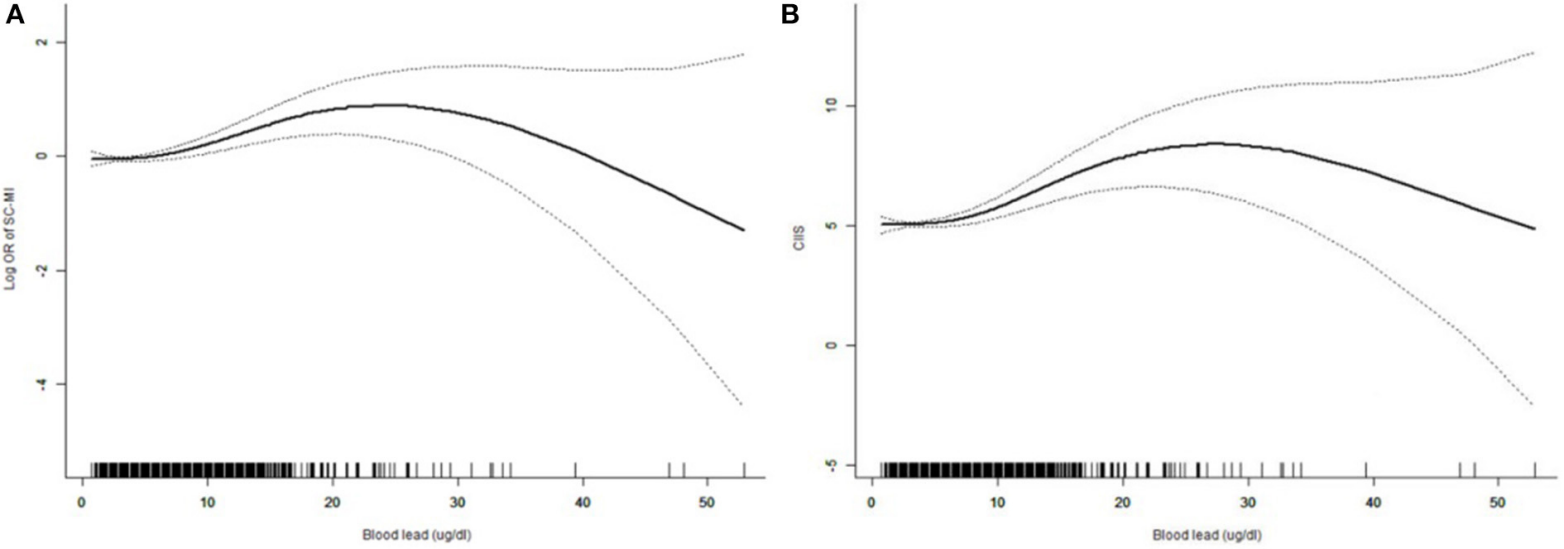
Restricted cubic spline plots of the association between blood lead with SC-MI **(A)** and CIIS **(B)**. The association was adjusted for age, sex, race, diabetes mellitus, hypertension, systolic blood pressure, body mass index, waist-to-hip ratio, triglycerides, total cholesterol, creatinine, fasting plasma glucose, hemoglobin Alc. SC-MI, subclinical myocardial injury; CIIS, cardiac injury/infarction score.

The restricted cubic spline curve showed that there was a non-linear correlation between blood lead and SC-MI (Figure 3). We further analyzed the threshold effect between blood lead and the prevalence of SC-MI (Table 3). Fitting 1-line and 3-piecewise logistic regression model to examine the relationship between blood lead and SC-MI. The results showed that the 3-piecewise logistic regression model was better than the 1-linear (*P* for log likelihood ratio test = 0.028). We determined that the inflection point of blood lead was 3.8 ug/dl. When the blood lead level ≥3.8 ug/dl, there was an independent positive correlation between blood lead level and the risk of SC-MI (OR 1.031, 95% CI [1.009, 1.053]; *P* = 0.006).

**Table 3:** Threshold effect analysis of blood lead on SC-MI using piecewise binary logistic regression models.

	**Model**	**Inflection point**	**Group**	**OR (95% CI)**	***P* for log likelihood ratio test**
Lead	Three-piecewise^a^	3.8 ug/dl	<3.8	0.939 (0.872, 1.011)	0.028
			≥3.8	1.031 (1.009, 1.053)*	
	One-line^b^	NS	NS	1.018 (0.999, 1.037)	

Analyses was adjusted for age, sex, race, smoking status, diabetes mellitus, hypertension, systolic blood pressure, body mass index, waist-to-hip ratio, triglycerides, total cholesterol, creatinine, fasting plasma glucose, hemoglobin Alc.

^a^Three-piecewise logistic regression model.

^b^One-line logistic regression model.

^*^P < 0.01, ^**^P < 0.001; SC-MI, subclinical myocardial injury; OR, odd ratio; CI, confidence interval.

### Subgroup analyses

**Figure 4:**
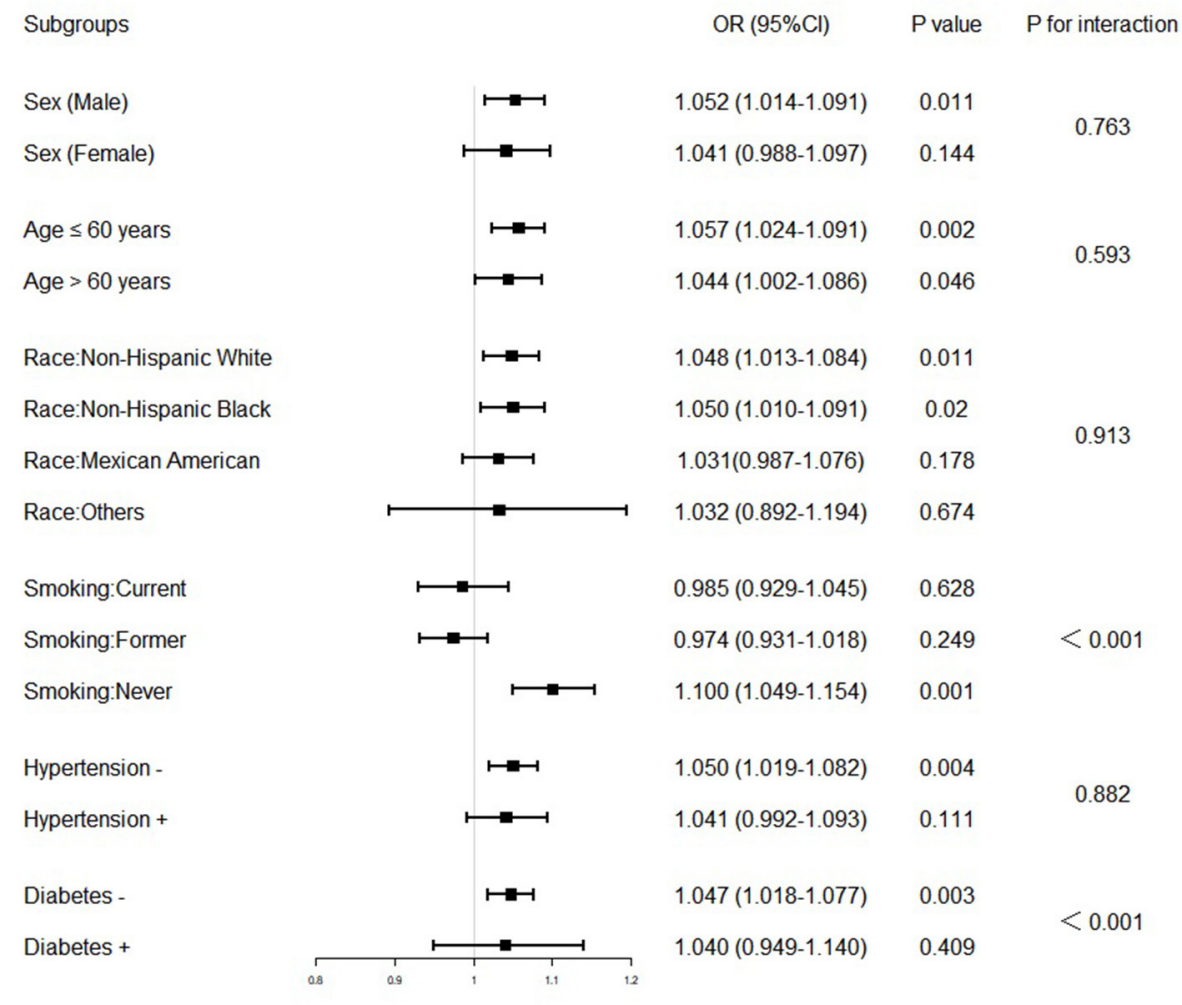
Association between blood lead and subclinical myocardial injury in various stratifications. The model used in the subgroups analysis consisted of all covariates used in Model 3 except for the variables that were used for stratification. The OR was examined by per 1-unit increase of blood lead. The interaction of blood lead and variables used for stratification was examined by likelihood ratio tests. OR, odd ratio; CI, confidence interval.

Although stratified analyses by sex, age, race, smoking status, hypertension, and diabetes confirmed that the association between blood lead and risk of SC-MI was stable in the subgroups of male, ≤60 years, >60 years, never smoker, non-Hispanic White, non-Hispanic Black or without hypertension and diabetes (*P* < 0.05), it was also unexpectedly found that the interaction between blood lead and diabetes and smoking status was significant (Figure 4).

## Discussion

As far as we know, this study was the first to confirm the relationship between lead exposure and SC-MI. The results showed that after adjusting for traditional cardiovascular risk factors, there was a non-linear correlation between blood lead and SC-MI, which was stable in the subgroups of male, ≤60 years, >60years, never smoker, non-Hispanic White, non-Hispanic Black or without hypertension and diabetes. Additionally, we also found the threshold effect of blood lead on SC-MI, that is, the blood lead level of ≥3.8 ug/dl was positively correlated with the risk of SC-MI independently.

At present, CVD is still the leading cause of death worldwide, and lead exposure accounts for 2% of the total burden of CVD, indicating that lead exposure may be an important potential risk factor for CVD (29). As some studies have shown, low or high levels of lead exposure were consistently associated with CVD, all-cause mortality and CVD-related mortality in different populations (including occupational and general populations) (2, 7, 13, 18, 30–32). For example, when using the health impact model for concentration response function analysis, Brown et al. found that approximately decline of 16–46% in CVD-related mortality from 1999 to 2014 could be attributed to a decline in blood lead levels (31). In addition, lead exposure is also dangerous in other non-fatal vascular diseases. For instance, Navas-Acien et al. found in a cross-sectional survey involving 2,125 participants aged ≥40 years that blood lead below safety standards remained strongly associated with the risk of peripheral artery disease after adjusting for confounding factors (20). Besides, Asgary et al. confirmed an independent association between blood lead and coronary artery disease in a case-control study matched by sex, age and place of residence (OR 1.050, 95% CI [1.009, 1.094]; *P* = 0.018) (17). Furthermore, recent studies have also found that blood lead was associated with an increased risk of carotid atherosclerosis and hypertension (19, 33). Besides clinical diseases, the side effects of blood lead were also observed in subclinical diseases, namely, several studies have demonstrated that blood lead was related to poor cardiovascular metabolic parameters, obesity, metabolic disorder and impaired left ventricular systolic function (12, 16, 34–38). In addition to blood lead, other studies have also revealed that urine lead and dietary lead intake were positively correlated with CVD risk factors, metabolic syndrome or all-cause mortality (39, 40). For humans, the evidence for the effects of lead exposure on the circulatory system is particularly extensive, while data on the relationship between lead exposure and SC-MI (a necessary pathway for CVD) are limited in the general population. However, our study showed for the first time the relationship between blood lead and the risk of SC-MI, and this relationship had a certain threshold effect.

Although our study confirmed the relationship between blood lead and SC-MI, the mechanism was still unclear. Currently, there are many mechanisms that may mediate the cardiotoxicity of lead. First, after summarizing the results of previous cellular, animal and human experimental studies, Vaziri showed that lead exposure could lead to endothelial injury, inhibit angiogenesis, hinder the growth and repair of endothelial cells, stimulate the proliferation and phenotypic transformation of vascular smooth muscle cells, and finally cause thrombosis, atherosclerosis, arterial stiffness, and even myocardial injury and CVD by promoting chronic inflammation and oxidative stress, interfering with signal transduction, increasing lipid peroxidation, limiting the use of nitric oxide, increasing endothelin production and enhancing adrenergic activity (22). Second, some epidemiological studies have found that lead exposure could lead to cardiovascular metabolic disorders, obesity and metabolic syndrome, which have previously been shown to be risk factors for myocardial injury (34, 35, 41). Third, lead exposure may also cause genetic and epigenetic changes through DNA methylation and histone modification. There is evidence that long-term chronic lead exposure is associated with abnormal DNA methylation in children, and this DNA methylation may mediate lead-related myocardial damage, which may genetically affect the occurrence of SC-MI (42–45). Nevertheless, more basic and clinical studies are needed to explore proven and potential mechanisms.

Although our research had achieved encouraging results, there were still several limitations. For example, as a cross-sectional study, we were unable to determine the causal connection between blood lead and SC-MI. In addition, bone lead is regarded as the best biomarker of long-term lead exposure, while bone lead is difficult to obtain in epidemiological and clinical studies. Besides, bone lead has a certain effect on bone metabolism, and bone metabolism is closely related to CVD and blood lipid levels (46, 47), so including bone mineral density (BMD) reflecting bone metabolism as a covariable in the study can reduce the deviation of the results and increase the stability of the results. However, as far as we know, BMD was only detected in the NHANES survey in 2001–2002 and 2005–2020, while the outcome variable of our study, SC-MI, was only detected in the NHANES survey from 1988 to 1994, so our study population only came from the participants who participated in the 1988–1994 NHANES survey, which meant that the participants in this study did not test BMD. Therefore, to sum up, we can not analyze BMD as a co-variable and there might be a certain bias in using blood lead as an alternative marker of lead exposure in this study. Moreover, there might be other uncontrolled confounding factors, such as diet. Finally, this study only included American adults, not teenagers and children, so there might be some limitations in extending the results to other countries and populations.

## Conclusion

In summary, our study showed a link between lead exposure and SC-MI, adding evidence for the potential myocardial damage effect of lead in CVD. Nevertheless, further cellular, animal and human studies are warranted to identify their causal relationship.

## Data availability statement

The original contributions presented in the study are included in the article/supplementary material, further inquiries can be directed to the corresponding authors.

## Ethics statement

The protocol was approved by the National Center for Health Statistics of the Center for Disease Control and Prevention Institutional Review Board. Written informed consent was acquired from all participants.

## Author contributions

ZW conceived and designed the study. XH and JL were responsible for the management and retrieval of data, contributed to initial data analysis, and interpretation. ZW drafted the initial manuscript. NL and QW revised the manuscript and were the guarantors of this work and had full access to all the data in the study and take responsibility for its integrity and the accuracy of the data analysis. All authors read and approved the final manuscript.

## Conflict of interest

The authors declare that the research was conducted in the absence of any commercial or financial relationships that could be construed as a potential conflict of interest.

## Publisher's note

All claims expressed in this article are solely those of the authors and do not necessarily represent those of their affiliated organizations, or those of the publisher, the editors and the reviewers. Any product that may be evaluated in this article, or claim that may be made by its manufacturer, is not guaranteed or endorsed by the publisher.
